# Simulation Experiment of Environmental Impact of Deep-Sea Mining: Response of Phytoplankton Community to Polymetallic Nodules and Sediment Enrichment in Surface Water

**DOI:** 10.3390/toxics10100610

**Published:** 2022-10-14

**Authors:** Rimei Ou, Lei Cai, Jinli Qiu, Hao Huang, Danyun Ou, Weiwen Li, Fanyu Lin, Xuebao He, Lei Wang, Risheng Wu

**Affiliations:** 1 Third Institute of Oceanography, Ministry of Natural Resources P.R.C., Xiamen 361005, China; 2 College of Marine Sciences, Shanghai Ocean University, Shanghai 201306, China

**Keywords:** polymetallic nodules, phytoplankton, growth rate, metal concentration, turbidity

## Abstract

In this paper, simulation experiments were conducted to study the response of phytoplankton biomass and community composition to the influence of polymetallic nodules and sediment at four stations in the western Pacific in 2021. Chlorophyll *a*, pico-phytoplankton cell abundance, and metal concentration were measured before and after 24 h of deck incubation. The results show that there were three different patterns of response, namely, restrained, stimulated, and unaffected patterns. The restrained pattern appeared in the filtered treatments at station Incub.01, and the stimulated pattern appeared in the unfiltered treatments at station Incub.02. The response of the phytoplankton was not detectable at stations Incub.03 and 04. Regardless, positive and negative responses were found in the dominant pico-phytoplankton group—*Prochlorococcus*—and with slight variation in *Synechococcus*. The concentration of manganese varied among the treatments compared to that of iron and other metals. The factors affecting the growth of the phytoplankton in this study were metal concentrations and turbidity. The phytoplankton biomass baseline may also have played an important role: the lower the biomass, the higher the growth rate. This study proved that deep-sea polymetallic nodule mining will have a specific impact on surface phytoplankton biomass, but turbidity and particle retention time could be important factors in mitigating the extent of the impact.

## 1. Introduction

As a more global, social economy has developed, the demand for metals has been triggered by the transformation of the energy structure. It is estimated that the demand for copper, manganese, cobalt, nickel, lithium, and other metals will continue to grow in the next few years in the clean energy technology industry [1]. However, the depletion of land-based metal mineral resources and the decline in metal mineral grade have greatly increased the interest in deep-sea mineral resources, where large repositories of these metals can be found with a high mineral grade [2].

Polymetallic nodules (PMNs) are an important deep-sea mineral resource rich in many key elements, including nickel, copper, manganese, cobalt, molybdenum, titanium, lithium, and rare-earth elements. PMN mining has the potential to play an important role in the transition from the global use of petroleum-based-energy to a low-carbon future, and it is also crucial for green energy, vehicles, and infrastructure, as well as for new technology and military applications [3]. There are four areas that are estimated to have a higher average abundance of PMNs, namely, the Indian Ocean (5 kg/m^2^), the Cook Islands (5 kg/m^2^), the Peru Basin (10 kg/m^2^), and the Clarion–Clipperton Zone in the East Pacific (15 kg/m^2^) [4]. These PMNs are primarily distributed in the abyssal plain and tropical oceanic basin at a depth of approximately 3500–6500 m, and these areas are characterized by a flat topography and slow flow and deposition rates, are low in primary production, and have a low seafloor biomass [5] with high biodiversity [6]. Therefore, PMN mining may have a significant impact on deep-sea ecosystems. Concerns about the environmental impacts of deep-sea mining are inevitable, as the density and diversity of most of the deep-sea ecosystem groups have undergone significant negative changes under simulated and tested nodule-mining disturbances. After the mining tests, very few faunal groups recovered to baseline or reference conditions after twenty years [7]. Moreover, the abundance, activity, and microbial organisms in sediments are still impaired 26 years after disturbances [8].

There have been many studies concerning the impacts of PMN deep-sea mining on the marine environment [9,10,11], and most of these studies have focused on the impact on benthic habitat destruction [12], sediment plumes [13], and the disposal of returning water [14,15]. Environmental impacts, including the fragmentation of benthic habitats, the loss of biodiversity and biomass, the disruption of buried carbon, pollution, metal toxicity, noise, and light, have been found in the sea-bed area or deep-sea water. However, we still lack solid evidence on the environmental impacts of deep-sea mining on the mesopelagic and euphotic layers. All mining of seabed mineral resources requires mining support vessels to return to the surface via lift pipes. Both the mineral extraction and trailing water discharge result in the discharge of slurry-containing nodules [16,17,18,19]. These slurries need to be dewatered prior to and after transfer, as the slurry-containing nodule ores and sediments spread to the upper ocean during this process, where metal particles, ions, nutrients, and clay particles have been predicted to impact the upper-layer water [7]. Although there are strict regulations surrounding the environmental impact of mesopelagic water bodies, especially in the euphotic layer [20,21], the accidental spillage or contamination of the slurry-mixed nodules is inevitable. This could cause significant interference to the euphotic ecosystem, characterized by the process of photosynthesis, whether through the enrichment of nutrients or turbidity [22].

As photoautotrophic organisms in the euphotic layer of the ocean, phytoplankton are the most important primary producers and account for nearly 50% of the primary productivity of the Earth’s biosphere, 85% of which occurs in oceanic regions [23]. The oligotrophic ocean is dominated by pico-phytoplankton, including *Prochlorococcus*, *Synechococcus*, and pico-eukaryotes, and these phytoplankton groups are sensitive to nutrients and metal concentrations [24,25,26]. Any form of nutrient and metal intervention, such as upwelling, eddy pumping, and anthropogenic activities, may lead to variations in the community structure of phytoplankton. In addition, phytoplankton are considered to be the core of the marine food web. Variations in the phytoplankton community affect the biogeochemical cycle in succession [27,28].

In terms of the effects of metals on phytoplankton, there are both positive and negative effects. Metals are essential for the growth of phytoplankton [29]. However, high concentrations may have toxicological effects [30]. Combinations of metal elementals have caused synergistic and antagonistic effects [31,32], which further complicate the direct effects on marine phytoplankton [33]. Despite the long-standing concerns of the potential environmental hazard to surface ocean phytoplankton caused by accidental spillage or contamination during PMN mining, the discussion should be supported by additional evidence. This is particularly important, as policies are being developed for the commercial mining of these deposits, and scientific evidence is urgently needed to guide these decisions.

In this study, we conducted on-deck incubation experiments to simulate the accidental spillage or contamination of slurry-mixed nodules during PMN mining to explore the possible acute, short-term effects on phytoplankton communities. The hypothesis was that phytoplankton biomass variation and community succession would occur as a result of accidental spillage or contamination. The present study aimed to (a) assess the effects of different culture media on the biomass of phytoplankton in an oligotrophic ocean and (b) to determine the key impact factors under the scenario of accidental spillage or contamination during PMN mining. Hence, this study could provide essential knowledge for the decision making of related interest groups and for the consideration of the impact of the spread of PMN mining slurry on phytoplankton in the open sea.

## 2. Materials and Methods

### 2.1. Study Area

**Figure 1:**
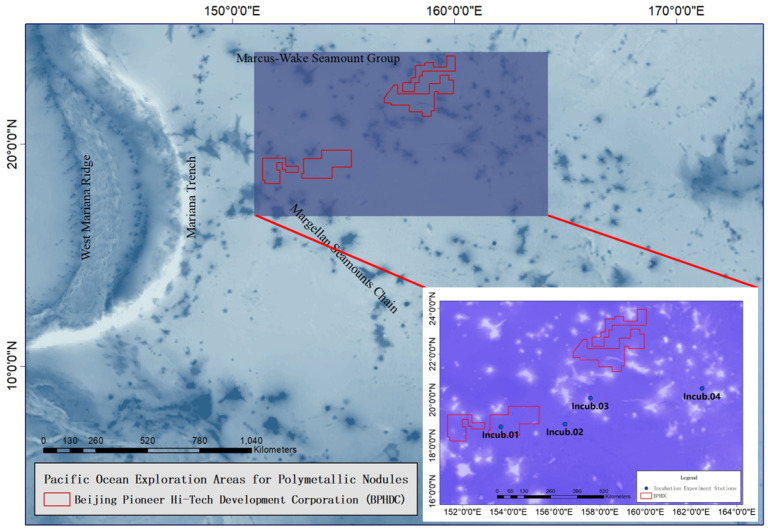
Map of the BPHDC’s PMN contract area and the location of incubation stations.

Sampling was conducted onboard R/V “Xiangyanghong 03” from 23 October to 2 November 2021 during the DY69 cruise organized by Beijing Pioneer Hi-Tech Development Corporation (BPHDC, Beijing, China) in the Northwest Pacific Ocean (153.673° E–162.480° E, 19.094° N–20.504° N, Figure 1 and Table 1). Four experiments were conducted at stations Incub.01, Incub.02, Incub.03, and Incub.04. Incub.01 was in the western PMN exploration licensed area, the M2 block of BPHDC. The other three stations were located on the east of the M2 block and almost at the same latitude.

### 2.2. Culture Medium and Experimental Design

One to two pieces of PMNs were ground to a fine powder, with a particle size of no more than 200 µm, through mesh. Moreover, 50 g of sediments in wet weight was also filtered through the mesh. Filtration was then mixed with particle-free water, which was prepared using an AcroPak™ 0.22 µm-pore-size capsule membrane. There were three kinds of culture media, which were prepared 12 h before the experiment, inside a 10 L Nalgene™ narrow-mouth, round Carboy with a spigot. The three culture media were composed of the following: (1) crushed PMNs and 10 L of surface seawater in a 10 L Nalgene carboy bottle; (2) sediment and 10 L of surface seawater in a 10 L Nalgene carboy bottle; and (3) crushed PMNs, sediment, and 10 L of surface seawater in a 10 L Nalgene carboy bottle.

**Figure 2:**
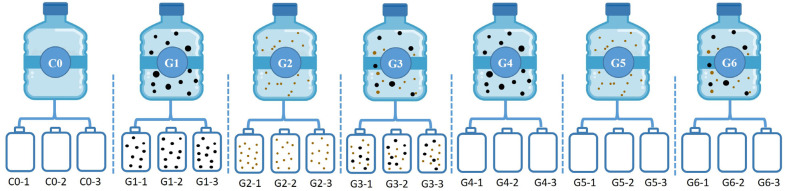
Schematic diagram of experimental design.

The experiment was divided into six treatment groups (G1–G6) and one control group (C0), and three duplicate samples for each group were determined. For each experiment unit of the treatment groups, 2.4 L Nalgene™ square PETG media bottles were filled with 2 L original surface seawater and 0.4 L culture medium. The control was three bottles filled with seawater without the addition of any culture medium. The groups G1–G3 had PMN culture medium, sediment culture medium, the mixed culture media of PMNs, and sediment, and the G4–G6 groups had the three kinds of culture media after filtration through an AcroPak™ 0.22 µm-pore-size capsule membrane (Figure 2). The specific settings for each treatment group were as follows:

Control group C0, no culture medium;

Group G1, add crushed PMN water medium;

Group G2, add sediment water medium;

Group G3, add crushed PMNs and sediment water medium;

Group G4, add crushed PMN filtered water medium;

Group G5, add sediment filtered water medium;

Group G6, add crushed PMNs and sediment filtered water medium.

After the seawater of each experimental group and the control group was mixed with the culture medium, all the experimental units were incubated on deck in the chamber under surface water temperature and available light for 24 h. Then, the water was sampled to quantify the chlorophyll *a* concentration, pico-phytoplankton abundance, and trace metal concentration.

### 2.3. Sampling and Measurement

#### 2.3.1. PMNs and Sediment

The PMNs and sediment used for the preparation of the water medium were sampled using a KC-Denmark box corer (Silkeborg, Denmark). For each incubation, one to three pieces of PMN (equal in total wet weight) and approximately 50 g sediment were collected and immediately stored at 4 °C until the preparation of the water medium.

#### 2.3.2. Chlorophyll *a*

The incubation water was filtered through an Advantec 25 mm diameter GF-75 (with a pore size of 0.3 µm) filter using a Millipore Swinnex^®^ Filter Holder (Merck & Co., Inc., Rahway, NJ, USA), with a vacuum pressure of no more than 0.02 MPa. After filtration, the filter was stored at −80 °C until it was analyzed in the laboratory.

Chlorophyll *a* was determined using a fluorescence analysis [34]. After the filter was extracted in 90% acetone solution in the dark and at −20 °C for 24 h, it was determined using a Turner Design Trilogy fluorometer (Turner Designs, Sunnyvale, CA, USA). The measurement of chlorophyll *a* concentration was performed on a spectrofluorometer, with the excitation and emission wavelengths set at 430 and 670 nm, respectively.

#### 2.3.3. Pico-Phytoplankton

A total of 1.8 mL of cultured seawater samples was fixed with 20 μL of paraformaldehyde and then stored in a −80 °C freezer. The abundances of pico-phytoplankton, including *Prochlorococcus*, *Synechococcus*, and pico-eukaryotes, were determined using a Becton, Dickinson and Company FACSAria (Franklin Lakes, NJ, USA) flow cytometer (FCM) according to Olson et al. [35]. The samples were thawed in a water bath at 37 °C in the dark. A total of 1 mL of samples was absorbed into the flow analysis tube, and 10 μL standard fluorescent beads was added as an internal reference. The samples were evenly mixed with a vortex mixer, and the samples were measured using FCM. The abundance of pico-phytoplankton was determined using FCSExpressV7 software.

#### 2.3.4. Trace Metal

Water samples were placed in a 50 mL centrifuge tube from each culture bottle after being filtered through a 0.22 μm syringe filter, and then they were stored in the −80 °C freezer. Water sample pretreatment: ultrapure water and 1 mL of water samples containing heavy metal ions to be tested after thawing were added into a 15 mL centrifuge tube and then stored in a −4 °C freezer. We quantified the absolute concentrations of iron (Fe), manganese (Mn), cobalt (Co), cadmium (Cd), copper (Cu), nickel (Ni), lead (Pb), and zinc (Zn) using an Agilent ICP-MS7700x inductively coupled plasma mass spectrometer (Palo Alto, CA, USA).

#### 2.3.5. Data Analysis

The effects of different additives on the growth of phytoplankton were compared using one-way ANOVA when the data were normally distributed, followed by a Tukey’s or Duncan’s multiple comparisons posttest; otherwise, the Kruskal–Wallis non-parametric test was used. *p* < 0.05 was used as the significance level. All statistical analyses were performed using IBM SPSS Statistics 26.0. Origin 2022 (OriginLab Corporation, Northampton, MA, USA) was used for all column chart statistical analyses, and ArcGIS 10.7 was used for figure plotting.

## 3. Results

### 3.1. Phytoplankton Biomass and Community Composition Pre-Incubation, and Different Response Patterns to PMNs and Sediment Enrichment

In the oligotrophic North Pacific Subtropical Gyre (NPSG) region, the chlorophyll *a* concentration was low at the surface, ranging from 0.043 to 0.062 μg/L. The highest chlorophyll *a* concentration was observed at station Incub.04, and the lowest was observed at station Incub.02. This showed that, although the phytoplankton biomass was very low, there was a value fluctuation among the stations of approximately half s. Therefore, the variation in the biomass baseline determined the different responses of the enrichment experiment.

**Figure 3:**
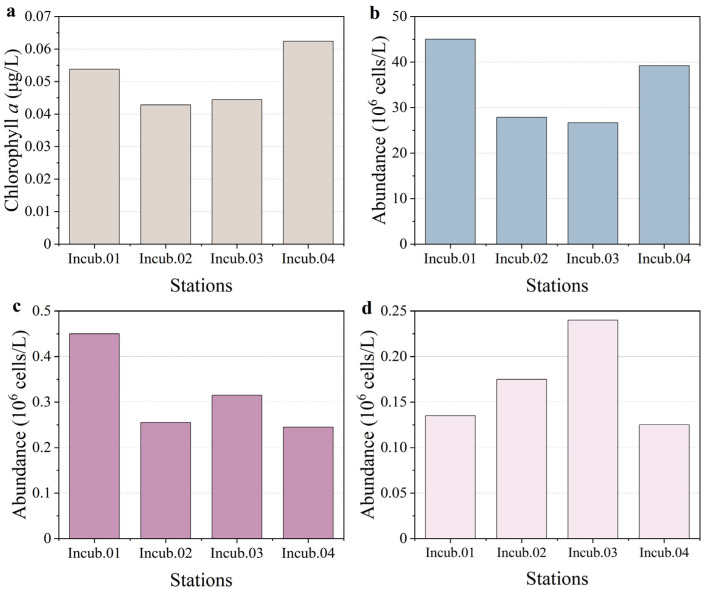
The pre-incubation (**a**) chlorophyll *a*, (**b**) *Prochlorococcus*, (**c**) *Synechococcus*, and (**d**) pico-eukaryote abundances at surface.

Pico-phytoplankton was the dominant community of phytoplankton in the NPSG region. The abundances of *Prochlorococcus*, *Synechococcus*, and pico-eukaryotes ranged from 26.650 to 45.025 × 10^6^ cells/L, 0.245 to 0.450 × 10^6^ cells/L, and 0.125 to 0.240 × 10^6^ cells/L, respectively. Overall, the highest abundances of *Prochlorococcus* and *Synechococcus* were observed at station Incub.01. However, the highest abundance of pico-eukaryotes was observed at station Incub.03, and the lowest was observed at station Incub.04. *Prochloroccus* was the dominant pico-phytoplankton group, and it was higher than the other groups of pico-phytoplankton at all the stations (Figure 3). Therefore, it could be observed that the cell abundance of pico-phytoplankton did not exactly correspond to the value of chlorophyll *a* concentration among the stations. On the one hand, this might be due to the variation in community composition; on the other hand, this might also be related to the physiological status of the cells.

**Figure 4:**
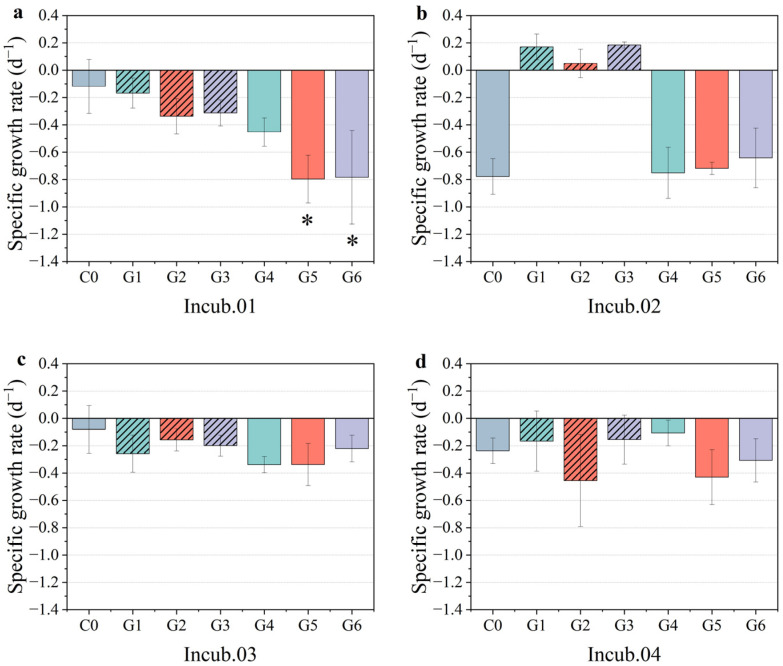
Specific growth rate at four incubation stations. (**a**) Restrained pattern at station Incub.01, (**b**) stimulated pattern at station Incub.02, and (**c**,**d**) unaffected pattern at stations Incub.03 and Incub.04. Green, orange, and purple denote the treatments of PMNs, sediment, and mixture, respectively. The unfiltered medium column is hashed, and the filtered is in pure color. * means that the difference between treatments and control group is significant at the 0.05 level.

According to the specific growth rate calculated by chlorophyll *a*, there were three different response patterns among the stations. Firstly, a restrained pattern was observed at station Incub.01. The phytoplankton biomass appeared to have negative growth among the treatment groups (Figure 4a). The treatment group with the filtered sediment medium (G5) and the filtered mixture medium (G6) showed significantly restrained phytoplankton growth compared to the control group (*p* < 0.05). Secondly, a stimulated pattern was observed at station Incub.02 (Figure 4b). The unfiltered treatments (G1, G2, and G3) had a significant stimulating effect on the growth of phytoplankton. Although all the filtered treatments (G4, G5, and G6) showed no significant effect on the growth of phytoplankton compared to the control and unfiltered treatment groups, they acted in opposition to the positive scenario. Thirdly, an ineffective pattern was observed at stations Incub.03 and Incub.04. There were no significant fluctuations in the specific growth rate among all the treatments compared to the control (Figure 4c,d). Although there were higher or lower values in each treatment group, there were no significant differences between the treatment group and the control groups overall. The response of the phytoplankton growth rate was not consistent, even when the enrichment experiment was conducted at a relatively small horizontal scale. As previously mentioned, this may have been caused by both the biomass baseline and phytoplankton community composition.

### 3.2. Response of Pico-Phytoplankton Community

**Figure 5:**
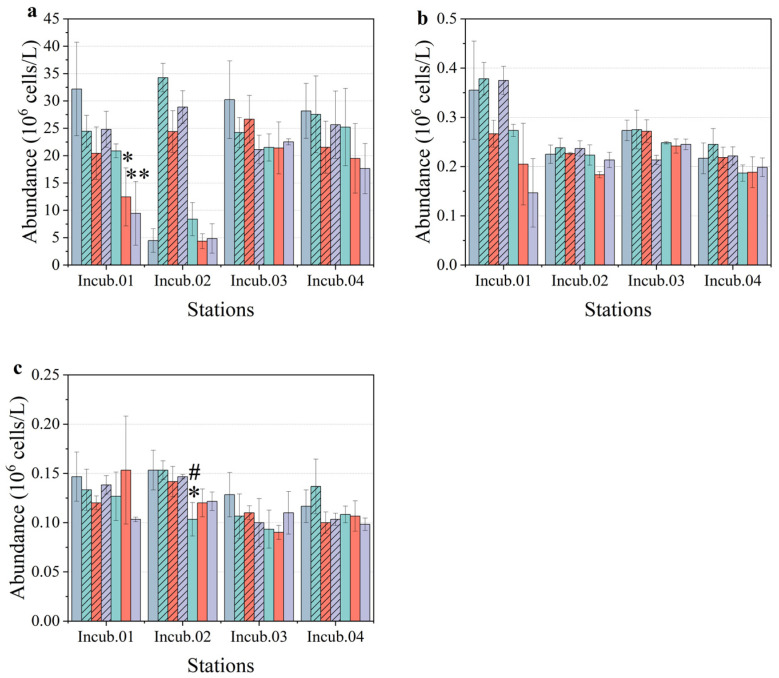
Cell abundances of (**a**) *Prochlorococcus*, (**b**) *Synechococcus*, and (**c**) pico-eukaryotes at incubation stations. Green, orange, and purple denote the treatments of PMNs, sediment, and mixture, respectively. The unfiltered medium column is hashed, and the filtered is in pure color. * and ** mean that the difference between treatments and control group is significant at the 0.05 level and 0.01 level, respectively; # means that the difference between the filtered treatments and unfiltered treatments is significant at the 0.05 level.

As the dominant phytoplankton in the oligotrophic NPSG region, the three fractions of pico-phytoplankton were ordered by cell abundance as *Prochlorococcus* > *Synechococcus* > pico-eukaryotes in this study. As mentioned above, the chlorophyll *a* concentration was the lowest at station Incub.02, and it was 30% lower than the highest value at station Incub.04. Corresponding to the lowest chlorophyll *a* concentration, the cell abundances of *Prochlorococcus*, *Synechococcus*, and pico-eukaryotes were 27.885 × 10^6^ cells/L, 0.255 × 10^6^ cells/L, and 0.175 × 10^6^ cells/L, which were 62%, 57%, and 73% of the highest values, respectively. However, the pico-phytoplankton community had a remarkable response to the enrichment treatments. Firstly, as a unique treatment group with a positive growth rate, the unfiltered treatment at station Incub.02 showed stimulation in all enrichment functions. Assessing the cell abundance of pico-phytoplankton, the abundance of *Prochlorococcus* at station Incub. 02 decreased by nearly 85% in the control group (4.473 ± 2.140 × 10^6^ cells/L) compared to pre-incubation (27.885 × 10^6^ cells/L) (Figure 3a and Figure 5a). A similar situation occurred in the filtered treatment groups, where the abundance of *Prochlorococcus* varied from 4.353 ± 1.361 × 10^6^ cells/L (G5) to 8.392 ± 3.029 × 10^6^ cells/L (G4). However, the scenario was the opposite for the unfiltered treatments. The abundance of *Prochlorococcus* increased dramatically with the unfiltered enrichment of PMNs and sediment, with the value varying between 24.393 ± 3.831 × 10^6^ cells/L (G2) and 34.262 ± 2.597 × 10^6^ cells/L (G1). As compared to the significant increase in the cell abundance of *Prochlorococcus*, the cell abundances of *Synechococcus* and pico-eukaryotes were nearly equivalent to that of the control group (Figure 5b,c). Therefore, although there was no significant difference between the control group and the other treatment groups in *Prochlorococcus*, it could be concluded that the stimulation of pico-phytoplankton growth by the unfiltered treatments at station Incub.02 had contributed to the response of *Prochlorococcus*.

Secondly, the cell abundance of *Prochlorococcus* in the filtered treatments decreased significantly at station Incub.01, which could explain the restrained effect on the growth of pico-phytoplankton (Figure 5a). The abundance of *Prochlorococcus* at station Incub.01 was 32.173 ± 8.551 × 10^6^ cells/L in the control group, which was 71.5% of the pre-incubation value (45.025 × 10^6^ cells/L). The abundance of *Prochlorococcus* decreased slightly in the filtered treatment, with values between 20.428 ± 4.809 × 10^6^ cells/L (G2) and 24.807 ± 3.277 × 10^6^ cells/L (G3). Although the abundance of *Prochlorococcus* in the filtered treatment of PMNs (G4) followed the same pattern as that of the unfiltered group, the other two treatments of the filtered groups represented a significant decrease in cell abundance (*p* < 0.01): 12.448 ± 5.311× 10^6^ cells/L in the filtered sediment treatment (G5) and 9.440 ± 5.814 × 10^6^ cells/L in the filtered mixture treatment (G6). Besides *Prochlorococcus*, the abundance of *Synechococcus* also decreased in these two treatments (Figure 5b). The cell abundance of *Synechococcus* was 0.205 ± 0.083 × 10^6^ cells/L in the filtered sediment treatment (G5) and 0.147 ± 0.070 × 10^6^ cells/L in the filtered mixture treatment (G6), which was lower than that in the control group (0.355 ± 0.010 × 10^6^ cells/L). For the pico-eukaryotes, there was no obvious response at station Incub. 01, but there was a remarkable restrained effect in the filtered treatment of PMNs (G4) at station Incub.02 compared to the control group and the unfiltered treatment (G1) (Figure 5c) (*p* < 0.05).

**Figure 6:**
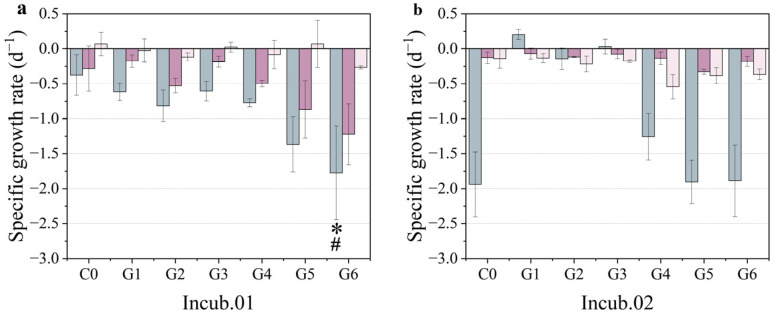
Specific growth rate at two incubation stations: (**a**) station Incub.01 and (**b**) station Incub.02. The gray, modena, and pale purple denote *Prochlorococcus*, *Synechococcus*, and pico-eukaryotes, respectively. * means that the difference between treatments and control group is significant at the 0.05 level, and # means that the difference between the filtered treatments and unfiltered treatments is significant at the 0.05 level.

Concerning the specific growth rate of pico-phytoplankton, it was similar to cell abundance. The filtered treatments with PMNs and sediment showed significant restrained growth of *Prochlorococcus* and *Synechococcus* compared to the control group at station Incub.01 (*p* < 0.05) (Figure 6a). In addition, enhancement occurred in the unfiltered treatments at station Incub.02 (Figure 6b). In the unfiltered treatment of sediment (G2) at station Incub.02, *Prochlorococcus* showed negative growth, while in the unfiltered treatments of PMNs (G2) and the mixture (G3) treatment groups, it had a positive increase. In addition, in terms of cell abundance, the unfiltered treatment of sediment showed a more obvious restrained or relatively mild stimulation in growth. Therefore, it could be inferred that the turbidity caused by the sediment was a key stress factor for the growth of *Prochlorococcus* as compared to the nutrients and metals introduced solely by clean PMNs. However, the filtered treatment showed a stronger restraint in growth despite the sediment-induced turbidity being eliminated, so we speculated that this process could be a balance between the positive effects of absorbed nutrients and metals and the negative effects of turbidity interference.

### 3.3. Metal Concentrations

**Figure 7:**
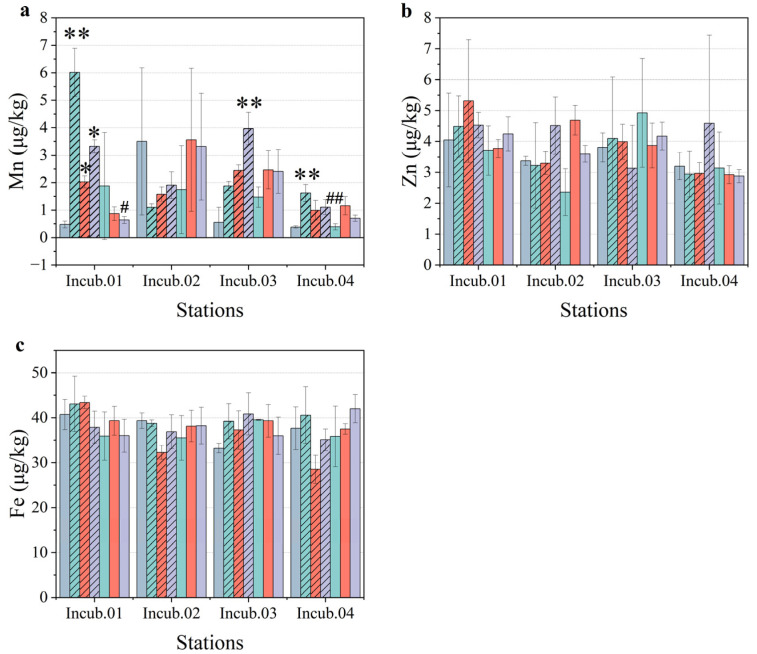
Metal concentration of (**a**) manganese, (**b**) zinc, and (**c**) iron at incubation stations. Green, orange, and purple denote the treatments of PMNs, sediment, and mixture, respectively. The unfiltered medium column is hashed, and the filtered is in pure color. * and ** mean that the difference between treatments and control group is significant at the 0.05 level and 0.01 level, respectively; # and ## mean that the difference between the filtered treatments and unfiltered treatments is significant at the 0.05 and 0.01 levels, respectively.

There were some significant differences between the unfiltered treatments and the filtered treatments, so the details were analyzed to determine the response mechanism. Firstly, as a unique treatment group with a positive growth rate, the unfiltered treatment at station Incub.02 showed stimulation in all the enrichment functions. Among the four incubation stations, the dissolved manganese concentration in the control group at station Incub.02 (3.499 ± 2.678 μg/kg) was much higher than that at the other three stations (0.475 μg/kg at Incub.01, 0.554 μg/kg at Incub.03, and 0.385 μg/kg at Incub.04) (Figure 7a). While the dissolved manganese concentration decreased in the unfiltered treatments at station Incub.02, it was 1.102 ± 0.131 μg/kg for PMNs, 1.576 ± 0.264 μg/kg for sediment, and 1.907 ± 0.485 μg/kg for the mixture of PMNs and sediment. However, the filtered treatment of sediment (3.556 ± 2.604 μg/kg) and the filtered mixture group (3.309 ± 1.943 μg/kg) maintained the same level as that of the control group. In terms of iron and zinc, there were no differences among the treatment groups (Figure 7b,c). At the same time, there were no significant differences between the control group at station Incub.02 and the control groups at the other stations. This implies that manganese absorption occurred, which induced growth in the pico-phytoplankton.

Secondly, there were some treatments that showed a restraint in manganese absorption, including the unfiltered treatments of PMNs at Incub.01 (6.014 ± 0.874 μg/kg), the unfiltered treatments of sediment at Incub.01 (2.025 ± 0.217 μg/kg), the unfiltered treatments of the mixture at Incub.01 (3.321 ± 0.238 μg/kg), the unfiltered treatments of the mixture at Incub.03 (3.964 ± 0.594 μg/kg), and the unfiltered treatments of PMNs at Incub.04 (1.620 ± 0.310 μg/kg) (Figure 7a). It could be observed that these treatments were all unfiltered treatments. Furthermore, restraint occurred more frequently in the treatment with added PMNs than in the treatment with added sediment. This implies that the restraint effect of PMNs on the growth of pico-phytoplankton may be stronger than that of sediments.

The response of the specific growth rate of the pico-phytoplankton community to metal concentration was compared in order to analyze the relationship between phytoplankton growth and metals. At station Incub.01, the specific growth rate of *Prochlorococcus* decreased when the dissolved manganese concentration was higher. In the filtered treatment groups, compared to the control group, the addition of the filtered medium had a stronger restrained effect on *Prochlorococcus* (*p* < 0.05). It could be speculated that the nutrients carried by the slurry played a critical role in promoting *Prochlorococcus* growth. The specific growth rate of *Synechococcus* in the filtered treatment of the mixture was significantly different from that of the control group and the unfiltered treatments at station Incub.01 (*p* < 0.05). However, it should be noted that the filtered treatment caused the removal of nutrients, including manganese, which had a more restrained effect on *Synechococcus*. The specific growth rate of *Synechococcus* in the filtered treatment was lower than that in the unfiltered treatments, although the difference was not significant (*p* > 0.05). There was no significant difference found for the remaining groups. Although there were no significant differences found in cell abundance and the specific growth rate of pico-eukaryotes between the filtered treatments and the other treatments at station Incub.02, the specific growth rate of pico-eukaryotes in the filtered treatment group was higher than that of the control group and the unfiltered treatments. Furthermore, the cell abundance of pico-eukaryotes was less than that of *Synechococcus*.

## 4. Discussion

Chlorophyll *a* concentration in ocean surface water is regulated by a complex interaction of physiological processes and ecological parameters. As the most important primary producers in a marine ecosystem, phytoplankton biomass and carbon fixation are significantly affected by light and nutrient supply. A diverse network of the available nutrients and physiological adaptation could be responsible for the successive reactions of the phytoplankton community [36,37]. In particular, in tropical and subtropical oligotrophic oceanic surface water, although there is sufficient light all year round, due to the scarcity of nutrients, the phytoplankton biomass and primary productivity are very low, and there are so few particles, ensuring that the turbidity is very low [38]. In addition, another characteristic of an oligotrophic ocean is the lack of metal elements, such as iron and manganese, which are essential elements for phytoplankton nutrient assimilation and carbon sequestration [39]. Overall, light, turbidity, nutrients, and metal concentrations are the major factors affecting the phytoplankton biomass and community structures in an oligotrophic ocean. The accidental spillage or contamination of slurry-mixed nodules during PMN mining affects these factors to a greater or lesser extent. However, according to the results of the present study, this effect is not exclusively negative. There was no high-impact scenario, and it may even promote the growth of phytoplankton under some conditions. The underlying cause may have been related to the basic phytoplankton biomass and community structures; however, it may have also been related to the balances among nutrients, metal concentrations, turbidity, and other factors.

**Figure 8:**
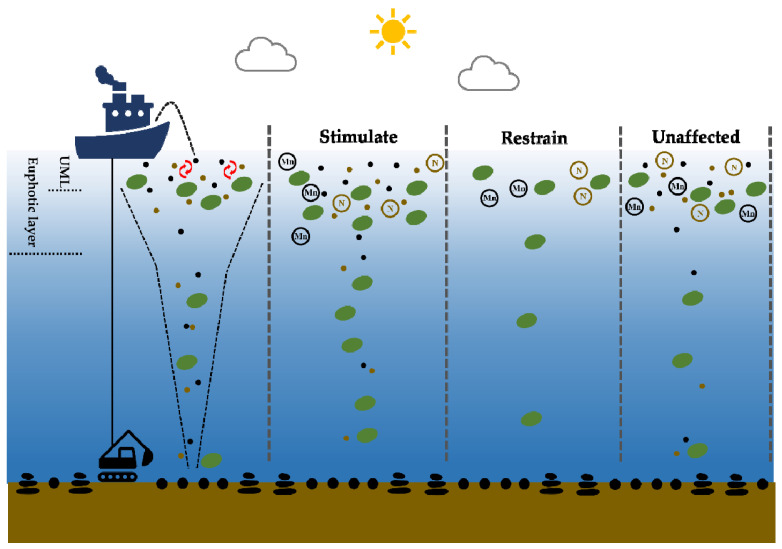
Conceptual figure of the three response patterns of phytoplankton to the PMNs and sediment associated with manganese, nutrients, and turbidity. UML denotes the upper mixing layer and is indicated by the red arrows. The black dashed line denotes the particle export.

In the present study, we detected the dissolved concentrations of eight metal elements, namely, iron, manganese, copper, cadmium, lead, zinc, cobalt, and nickel, among which five metal elements, namely, copper, cadmium, lead, cobalt, and nickel, were below the detection limit. Iron and manganese are two important trace metals required by phytoplankton, and the availability of iron limits approximately one-third of the primary productivity of the global ocean [40,41]. The availability of manganese also plays a limiting role in the growth of phytoplankton [42]. In this study, we only observed an obvious fluctuation in manganese concentration among the treatments, accompanied by the response of phytoplankton biomass and pico-phytoplankton cell abundance. However, there were no obvious variations in the iron concentrations among the treatments. It was difficult to diagnose whether there was a synergistic pattern between the concentration of dissolved iron and manganese. However, there were still some interesting phenomena at individual stations. For instance, at station Incub.03, the concentration of dissolved iron was the lowest in the control group, the concentration of dissolved manganese was also very low, and the biomass of pico-phytoplankton increased positively, while the metal concentration increased in varying degrees after the addition of different treatments, and the higher the concentration, the more obvious the restrained effect. The bioavailability of iron is the most significant factor for *Synechococcus* growth, as was found in a previous study, which also implied its significance for pico-phytoplankton in an oligotrophic ocean [43]. The concentration of dissolved manganese in the control group was much higher than that in the other treatments at station Incub.02, and the biomass was much lower than that in the other groups. The flexibility of the manganese quota in phytoplankton was relatively limited and was approximately 2.7 times that of iron [44]. According to previous studies, phytoplankton require a higher manganese concentration to grow under low light intensity [45,46]. This could explain why only the concentration of manganese produced a response in the present study, predominantly in the unfiltered treatment groups. This suggests that the effect of PMN mining on the growth of phytoplankton had a coupling effect with metal and turbidity (Figure 8).

Variations in turbidity caused by particulate matters have previously been found to affect the growth of phytoplankton [33,47]. The response of phytoplankton to suspended sediment showed an increase in biomass at station Incub.02, which could indicate that the suspended sediment stimulated the increase in phytoplankton growth as a result of nutrient and metal release. As compared to the control group, the increase in turbidity caused by suspended particles generally slowed down biomass productivity and the rate of light adaptation. In addition, there was a decrease in the phytoplankton growth rate at most stations. The removal of particles in the filtered treatments did not have any significant effect on biomass. This indicates that there could be another mechanism with a positive correlation between the retention time of PMNs and sediment particles in water and the concentration of the release. Therefore, the unfiltered treatment provided an opportunity to observe the PMNs and sediment, and the subsequent available nutrients and metal concentrations for phytoplankton were significantly higher than those in the filtered treatment. Furthermore, Burns (1980) showed a pattern of stimulating phytoplankton growth. Nutrient and metal impacts were identified between the positive effect of metal concentration and the negative effect of turbidity (Figure 8).

Changes in oxygen or hydrogen sulfide concentration, temperature, salinity, and dissolved substances caused by the release of material from deep-sea mining activities have caused changes in water quality [48]. In a previous study, Burns (2008) showed that an increased particle concentration in the plume caused a marked reduction in the amount of light penetrating the upper mixed layer, but the shielding effect of the suspended sediment was transient due to the rapid sinking rate of the particles [49]. However, in the present study, as the PMNs and sediments were added in bottles for 24 h incubation, the retention time of the particles was prolonged, thus amplifying this effect. From the view of the actual engineering of deep-sea mining, it has been estimated that the volume of water at 5 m to be transported past the mining vessel would contain 9 ppm effluent in one year. Therefore, it may be unlikely that a measurable effect on productivity, oxygen, and nutrient concentration would result from this kind of mining activity [50]. Primary production affected by deep-sea mining usually depends on the interaction among several variables. It is dependent not only on the rate, volume, and composition of the spread but also on the rates of dilution and mixing. For example, phytoplankton biomass has been moderately reduced by the release of crashed deep-sea polymetallic Fe–Mn crusts. In the short term, phytoplankton biomass and community structure in an oligotrophic open ocean are dynamic, but the potential impact of their release is low due to the reactivity of coarse-grained ore materials in terms of sedimentation and the low surface area [33].

## 5. Conclusions

To date, the possible effects and underlying mechanisms of PMN mining on marine phytoplankton have not been fully discussed. This study provided simulated experimental data on the response of phytoplankton to slurry emissions from PMN mining and explored the possible environmental impacts. Future research on the effects of deep-sea mining on pelagic marine life and its role in marine biogeochemistry is much needed. In this study, the following results were found:

1. There were three different response patterns of phytoplankton to the addition of PMNs and sediments, namely, restrained, stimulated, and unaffected patterns. There was a significant variation in our results.
2. The major factors that affected the response mechanism were complicated and acted in coordination. The phytoplankton biomass baseline determined the response, which indicated that a lower biomass was more likely to positively affect growth. Manganese assimilation was the most important physiological characteristic of pico-phytoplankton, especially for the dominant *Prochlorococcus*.
3. The turbidity caused by the sediment was another important factor in the response of phytoplankton. As compared to the negative effect of reducing the available light, the unfiltered particles provided more available nutrients and metals for the growth of phytoplankton.

However, it is important to recognize that the simulated experimental data in the present study were the result of short-term cultivations. Research methods closer to those used in the present discharge process and studies over longer time periods should be developed to explore the response of phytoplankton to deep-sea PMN mining.

## Figures and Tables

**Table 1:** Station information for in situ enrichment incubation experiments on board.

Station	Date	Time	Latitude (N)	Longitude (E)	Depth (m)
Incub.01	27 October 2021	09:41	18°49.8317′	152°53.3914′	5528
Incub.02	28 October 2021	07:43	18°49.9030′	153°16.2637′	5668
Incub.03	29 October 2021	04:43	18°49.6079′	153°40.4914′	5650
Incub.04	30 October 2021	00:27	18°33.7015′	153°40.5757′	5645

## Data Availability

Data are available on request to the authors.
